# The Study of Physical Diagnosis without a Master

**Published:** 1846-08

**Authors:** 


### EDITORIAL DEPARTMENT.

*The study of Physical Diagnosis without a Master.*—In the American Journal of Med. Science for July, 1846, we find a notice over the initials A. S. of several recent treatises on Physical Diagnosis, in which the following occurs:

"The very best of the treatises mentioned are almost worthless unless expounded by a living teacher, and illustrated at the bed-side. They are worse than worthless; they are pernicious: they lead to positive error. Unless the student be told what are the sounds he hears in auscultation, unless one more experienced than himself inform him of the name of every sound, and present to his ear the sound corresponding to every name; the descriptions and definitions of his "Manual" must continue meaningless to him; or if, by chance or skill, he now and then succeeds in rightly coupling the characters of a rhonchus with its corresponding name, he can never feel assured of the fact until it is corroborated by a living teacher. He is as little qualified to interpret acoustic signs, as one who has learned to read a foreign language by the help of dictionary and grammer, is to translate that language when he hears it spoken. Those who have honestly made the experiment of learning auscultation through books alone, will be the very first to admit the justness of these views. For the beginner, clinical instruction in auscultation is essential. The more he listens, and the less he reads, the surer and more rapid will be his progress. When once his ear is familiarized to the new language, he may study with advantage its theory; indeed he *must* then study it in order to perceive the principle which pervades the phenomena he has witnessed, and to acquire the power of analyzing and interpreting them in whatever new combinations they may occur."

We make this quotation in order to dissent from the opinion therein expressed. We believe that such views, advanced by those who are supposed competent to speak with authority on the subject, have done vastly more to retard the study of physical diagnosis than any difficulties inherent in the pursuit, or any other cause whatever; and we should be glad to contribute our humble efforts toward the removal of an impression, which, in our estimation, is as gratuitous, as it is prevalent. We have no disposition to undervalue the importance of clinical teaching in this department. We admit that the direction of a competent instructor, and the opportunity of studying a large collection of cases, with that leisure, and freedom from restraint, which are afforded at public institutions, are invaluable to the medical student. He may acquire under these circumstances, in days, and weeks, what will require months, or years, if restricted to the opportunities of private practice, and the facilities afforded by elementary works. Clinical teachings, therefore, are by all means to be obtained, if practicable, and at places where ample illustrations and verifications are available—but are they a *sine qua non?* We will devote a little space to this inquiry. A person of decent intelligence, and manual tact, assuredly may qualify himself to practice percussion with the aid of a few *printed* directions. He may, if he be not superlatively obtuse, very soon learn to distinguish from healthy resonance, dullness and flatness of sound. Here is one half the battle gained, as every one conversant with the subject will admit. Close attention, long practice, and a peculiar delicacy of the acoustic sense, are doubtless necessary to enable the explorer to distinguish the slighter shades of comparative dullness on percussion, but for all the ordinary purposes of diagnosis, this is not important. The question is generally, are any portions of the chest distinctly dull, or flat, on percussion, and it is to be borne in mind that we always have the advantage of comparison with different parts of the same thorax. Now, suppose that we find distinct flatness. In 19 out of 20 cases the object will be to ascertain if it be due to the presence of fluid, to solidification from inflammation, or to tubercular deposit. But as cases of the latter are generally easily separable from the two former, it will follow that the object will be, in one class of cases, to distinguish between the fluid and the solidification; and, in the other class, to decide on the fact of tubercular deposit. We resort then to auscultation. In the great majority of cases, the presence of fluid, sufficient to occasion flatness, will be associated with absence of all respiratory murmur. How easy, then, with the help of circumstances which we need not here specify, to diagnosticate pleuritis with effusion, or empyema! but how often do practitioners who do not employ physical exploration, treat such cases without suspecting the real condition of the chest! If, on the other hand, the respiratory murmur be present, but altered in its character, being perhaps louder than natural, but, at all events, bronchial, tubular, or blowing, instead of vesicular, the case is one of solidification from inflammation or tuberculous deposit, as other circumstances appertaining to the case may enable us to determine. Now to be able to know whether the respiratory murmur be present or not, and, if present, whether it be vesicular or tubular in its character, cannot be a very difficult accomplishment for any one to acquire with the help of descriptions in books, and a little common sense; and these, united to an acquaintance with the various pathological circumstances therewith connected, will suffice for the discrimination of the two diseases just mentioned, provided the pneumonitis have advanced to the second stage.

But suppose the point be to distinguish pneumonitis, in the first stage, from bronchitis, and other affections. The characteristic *rale* (the crepitant) we venture to assert may be recognised for the first time by nineteen out of twenty intelligent students or practitioners. The cases in which a fine capillary mucus *rale* is liable to be confounded with it, are so rare that they will not intefere with ordinary diagnosis; the possibility of confounding them may easily be recollected, and, also, the few and infrequent pathological conditions, beside pneumonitis, in which the crepitant *rale* may be present.

If the case be bronchitis—what are the facts required for the diagnosis? Good resonance on percussion; absence of, or diminished respiratory murmur, or more or less of the great variety of the sonorous, the sibilant, or mucus *rales*. These *rales* may be divided and subdivided to any degree of tenuity, but no stethoscopist will contend that these refinements are very important for most of the practical purposes of diagnosis.

We pass to tuberculosis of the lungs. The question in suspected cases are: Is the case one of tubercular disease, or is it bronchitis simply? Has tubercular deposit taken place, and to what extent? Are there cavities in the lungs? The first of these queries is perhaps the most difficult, in certain cases, of any which occur in the range of pectoral diagnosis. We do not wish to depreciate that acuteness of perception which will distinguish a slight dullness on percussion, and a slight roughness of the respiratory murmur, sufficient, in a very doubtful case, to turn the scale in favor of the diagnosis of tubercular disease. But such very doubtful cases are not frequent, and it is well known that in such cases the most experienced stethoscopists are liable to commit errors in trusting too confidently to this delicacy of hearing, and relying too exclusively to the physical signs. The two other enquiries present no greater difficulties than appertain to the other diseases mentioned. Dullness, or flatness on percussion over a greater or less space; absence of respiratory murmur over this space, or the tubular respiration; a distinct, circumscribed, blowing respiration, are not very obscure or abstruse signs to understand, and verify, with no other aid than descriptions, and a decent aptitude of comprehension on the part of the learner.

We have thus mentioned four pathological affections, viz. pleuritis with copious or purulent effusion, pneumonitis, bronchitis, and phthisis. Let it be considered how large a share of pulmonary diseases, in ordinary practice, are embraced in these four divisions; and in connection with this, how few and simple are the prominent points which relate to their physical exploration, and we cannot see why it should not be expected of practitioners who have not enjoyed the advantages of oral instruction, in large hospitals, that they should be able to practice this invaluable auxiliary to rational methods of diagnosis.

It would be easy to show that the physical signs of other, and more infrequent forms of pulmonary disease, are scarcely less easy of comprehension, and recognition, in so far as they concern ordinary practical purposes; but our object is not to attempt a compendium of practical rules in this province of our art, but only to adduce sufficient examples to illustrate the position we have assumed. If a person be competent to determine whether portions of the chest are relatively dull or flat on percussion; to distinguish whether the respiratory murmur be absent or present, or relatively feeble, or increased in intensity; to discriminate between tubular, or blowing, and the normal vesicular murmur; and to recognise the crepitant *rale*, and the more common varieties of the mucus, or bronchial *rales*, he is prepared, so far as an appreciation of the signs are concerned, for the satisfactory employment of this method in most of the cases with which he will meet in ordinary medical practice. And we contend that to acquire this knowledge is by no means a herculean task under any circumstances, provided the learner can find patients with the forms of disease in which the several signs are exemplified. The essential signs themselves are both few and simple. It is chiefly by needlessly minute classifications, and wiredrawn distinctions, that the subject has been made to appear too intricate to be studied, except by those who can devote long and exclusive attention to it, with peculiar facilities. This idea repels the majority of medical students and practitioners from any attempt to make it available in their own hands. Books and Lectures devoted to this subject *in extenso*, frequently do more harm than good by confirming this mistaken idea. Readers and hearers are confounded, and discouraged by the interminable divisions and subdivisions, the fine spun explanations, and cumbrous nomenclature. We are right glad to see evidences of improvement in these respects. We are told that M. Raciborski, formerly resident physician at La Charite, Paris, "is in the habit of delivering a complete course of clinical instruction in auscultation and percussion in about a dozen lectures." [O. W. Holmes, M. D., Dissertation on Direct Exploration.] And, of late, several treatises have been published which present the subject in its true simplicity. Indeed, in the views which we have expressed, we are sustained by the opinions of some of the most distinguished proficients in this department. Were it not so, we should feel a delicacy in enunciating them.

There is another consideration which we would adduce. Laennec, the discoverer of auscultation, certainly had neither the advantage of books, nor living teachers; yet, in a short period he ascertained the fundamental facts pertaining to the subject, which, for the most part, still remain substantially as determined by him. It must be admitted that it is not fair to estimate the abilities of medical students and practitioners, generally, by the genius of Laennec; yet it does seem fair to presume that a person with respectable talents for observation and acquirement, with even the aid of books alone, may follow the paths which Laennec discovered and traced without any guide. If it required a Columbus to discover a new continent, any mariner of tolerable skill, has, ever since, been able to cross the ocean and find it.

The truth is, unless we greatly err, it is not the difficulty of appreciating the physical signs, which is the chief obstacle in the way of employing them satisfactorily in diagnosis, but the great, and real difficulty is in the exercise of judgment and ratiocination in bringing them together; estimating their proper weight, separately and combined; and making use of them not to the exclusion of, but, in all cases, in conjunction with rational or vital symptoms. It is an easy thing, with or without a teacher, for the senses to get the sounds, but, when obtained, they are to be subjected in every case to processes of reasoning, like all other sensible facts. In short, it is with signs, as with symptoms, the mere observation of them, is one thing, and the application of sound logic to deduce from them just conclusions, is quite another thing. The success of the latter will depend on the ability, mental discipline, and experience of the practitioner. We have been betrayed into a greater length than we had intended when we commenced penning this article; and, as it is, we should be disposed to say much more on the subject if this were the occasion or place for an extended discussion. Our observations, and, we may add, experience have satisfied us that the reason why practitioners generally, old and young, do not avail themselves of the invaluable aid in diagnosis to be derived from physical exploration of the chest, is because it is imagined to be a vast and difficult undertaking, and not to be attempted except at Paris, or some of the larger cities, under the personal direction of living teachers, and in extensive hospitals. We are also satisfied that this impression is entirely erroneous, and that every practitioner, old and young, residing in cities or villages, may, if they choose, with a proper effort, sufficiently qualify themselves to appreciate physical signs, at least, to a degree which they will never consent to dispense with, when once they have commenced the acquisition; and to apply to the facts therefrom derived the same powers of judgment and reasoning which they are capable of bestowing upon facts derived from other methods of observation. Entertaining these views, as we have said, we should be rejoiced to do what we can to remove such impressions from the minds of those who have, in consequence, hitherto neglected percussion and auscultation. And should our few remarks happen to excite among any of the class referred to, the determination to act upon the views which have been expressed, we have no fears that the results will not confirm their correctness. We say, then, let students and practitioners, if they can, by all means, avail themselves of clinical instruction, and of public institutions; but if it be not practicable to obtain these advantages, the end is still attainable. With the help of a good elementary treatise which presents the subject in its true simplicity, and with such illustrations as almost every village will furnish, satisfactory progress may be made. Greater effort and longer time will be necessary, but enough may be accomplished to secure an advantage in diagnosis, which, when once enjoyed, the possessor would sacrifice the profession rather than dispense with. The work which, without any invidious distinction, we would take the liberty of recommending to the beginner, is that by Dr. Gerhard, on the *diagnosis, pathology and treatment of diseases of the chest.* The great recommendation of this work is, that it treats of physical diagnosis divested of needless refinements and elaborate mystification; and, at the same time, enforces, and illustrates the point that the great value of this method is not in its detached facts, but in the processes of ratiocination of which these facts are necessary elements or data. In addition to this, it seems to us a great advantage to treat of the physical signs conjunctively with symptoms. This is a distinguishing feature of Dr. Gerhard's work.

It is, moreover, not a reprinted English work with notes and additions, but a *bona fide* American book. We do not state this as a reason why it should be preferred if it had less merit than an imported publication, but as we conscientously believe it is not excelled, we may with propriety take additional satisfaction, on this account, in recommending it to the American reader.

In the preceding desultory remarks we have referred to physical diagnosis in its relations to pulmonary diseases. Diseases of the heart are usually regarded by non-auscultators as even more removed, as regards their physical signs, from ordinary capacities and self-education. The senior practitioner, or he who has not enjoyed the opportunities of studying with the aid of a master, if he does not distrust the whole subject, at least is well persuaded that it will never be of any advantage to him. It may do, he says for the young men, and those who visit Paris, but it is useless for him to think of looking at all into it. Here lies the rub. Could all think otherwise, and feel that it was a subject from which, with a little attention, they might derive great assistance, even without leaving their homes and business for the express purpose of spending a season in Paris or Philadelphia, to be initiated into the wonderful mysteries of the stethoscope, we believe that instead of being limited to a few, the study of the physical signs would be *at once* very generally prosecuted. The quotation which served as the text for our remakrs, applies of course, equally to the physical exploration of heart diseases. The late Dr. Hope, however, whose competency to speak authoritively on any point connected with this subject will hardly be questioned, entertained quite a different opinion from the Reviewer in the American Journal. Speaking of the physical signs denoting the location of organic disease in the particular valves of the heart, he says : "I have no doubt that ere long the physical signs in particular will be universally admitted to be as simple and easy as I represent them to be, since I have found by trials that intelligent students are competent to make particular diagnoses after a verbal explanation not exceeding a quarter o an hour's duration." [On Diseases of Heart. Am. edition p. 374]. And in another place he states, that on one occasion he explained to four pupils, who had had no previous practical acquaintance with auscultation applied to the study of diseases of the heart, the general rules by which the particular seat of valvular disease may be diagnosticated, occupying only ten minutes in the explanation, and then intioduced them to six patients—presenting five varieties of valvular disease. They delivered, after their examinations, sixteen diagnoses, fourteen of which coincided with his own, and were therefore deemed correct. We regard this sufficient testimony in favor of our position in so far as diseases of the heart are concerned. Not to extend this article farther, we would say, in conclusion, that for the application of percussion and auscultation to be universally practiced by the profession, we believe it to be only necessary for their simplicity to be appreciated. Time was, not long since, when a clinical teacher could walk the wards of an hospital, using, in derision, the stethoscope as a bouquet-holder. But he who should now attempt a practical *jeau d'esprit* of this kind, would be deemed incompetent to hold a responsible public station. And the time will come when ridicule or neglect of these methods by the most obscure practitioner will be as inexcusable as they would be now in the public teacher. Deeply impressed with the importance of securing this end as speedily as possible, we regret to see either words or acts which tend to confirm the idea of mystery and difficulty, which, unfortunately, in the minds of a large portion of the profession, still invests the subject of physical diagnosis.

